# Influence of Parental Healthy-Eating Attitudes and Nutritional Knowledge on Nutritional Adequacy and Diet Quality among Preschoolers: The SENDO Project

**DOI:** 10.3390/nu10121875

**Published:** 2018-12-03

**Authors:** Andrea Romanos-Nanclares, Itziar Zazpe, Susana Santiago, Lucía Marín, Anaïs Rico-Campà, Nerea Martín-Calvo

**Affiliations:** 1 Department of Preventive Medicine and Public Health, School of Medicine, University of Navarra, 31008 Pamplona, Spain; aromanos@alumni.unav.es (A.R.-N.); izazpe@unav.es (I.Z.); ssantiago@unav.es (S.S.); aricoc@unav.es (A.R.-C.); 2 Department of Nutrition, Food science and Physiology, School of Pharmacy, University of Navarra, 31008 Pamplona, Spain; 3 Research Centre Network on Obesity and Nutrition (CIBERobn) Physiopathology of Obesity and Nutrition, Institute of Health Carlos III, 28029 Madrid, Spain; 4 IdiSNA, Navarra Institute for Health Research, 31008 Pamplona, Navarra, Spain; 5 San Jorge Primary Care Health Center, Servicio Navarro de Salud-Osasunbidea, 31008 Pamplona, Spain; lucia.marin.alcala@cfnavarra.es

**Keywords:** diet quality, preschool children, nutritional adequacy

## Abstract

Parental nutrition knowledge and attitudes play a fundamental role in their children’s food knowledge. However, little is known about their influence on their children’s diet quality and micronutrient intake. Thus, we aimed to assess the association of parental nutrition knowledge and healthy-eating attitudes with their children’s adherence to the Mediterranean dietary pattern and micronutrient adequacy. Parental healthy-eating attitudes and knowledge of the quality of their child’s diet as well as anthropometric, lifestyle, and nutrient intake characteristics were recorded with a basal questionnaire that included a 140-item-food frequency-questionnaire. A total of 287 pre-school children were included in the analyses. Intake adequacy was defined using the Estimated Average Requirements (EAR) cut-off point method. We developed a parental nutrition knowledge and healthy-eating attitudes scores and evaluated whether they were independently associated with 1) children’s inadequate intake (probability of failing to meet ≥3 EAR) of micronutrients, using logistic regression analyses, and 2) children’s diet quality (adherence to the Mediterranean Diet according to a Mediterranean Diet Quality Index for children and adolescents, the KIDMED index), using multiple linear regression models. A higher score in the parental healthy-eating attitudes score was associated with lower risk of failing to meet ≥3 EAR compared with the reference category (odds ratio (OR): 0.3; 95% confidence interval (CI) 0.12–0.95; *p* for trend: 0.037) and a higher adherence to the Mediterranean diet in the most adjusted model (*β* coefficient: 0.34; 95% CI 0.01–0.67; *p* for trend: 0.045). Our results suggest a positive association of parental healthy-eating attitudes with nutritional adequacy and diet quality in a sample of Spanish preschoolers. Public health strategies should focus on encouraging parental healthy-eating attitudes rather than simply educating parents on what to feed their children, recognizing the important influence of parental behavior on children’s practices.

## 1. Introduction

Healthy eating during childhood may be one of the most determinant factors in human health and it is a well-known growth and developmental booster [1]. Eating behaviors are shaped by intrinsic (genetic, age, and sex) and environmental factors, such as family, friends, or neighborhood [2]. Parents are important agents in the promotion of health, behavior, and education of their children; they create food environments and play a key role in structuring their children’s first experiences with food and eating through their own beliefs, food practices, perspectives, eating attitudes, knowledge, and understanding of the benefits of food and nutrients on health [3]. Particularly, parental nutrition knowledge and attitudes have been described as important factors for children’s healthy food knowledge [3]. However, little is known about children’s diet quality and micronutrient adequate intake in relation to their parents’ or caregivers’ attitudes.

Knowledge is a complex scheme of beliefs, information, and skills gained through experience and education [3]. In terms of nutrition and eating, knowledge can be described as the familiarization of the benefits of food and nutrients on health and the ability to remember and recall specific terminology and information on the subject. 

Eating attitudes are emotional, motivational, perceptive, and cognitive beliefs that influence the behavior or practice of an individual whether or not they have knowledge [4]. Attitudes are measured to identify individual positive or negative disposition regarding a health problem, dietary practices, nutritional recommendations, dietary guidelines, or dietary preferences. 

Dietary intake and diet quality are difficult to measure. Despite some limitations, Food Frequency Questionnaires (FFQs) are considered the most efficient and feasible method to assess usual dietary intake, whereas diet quality indices can be defined either *a priori* or *a posteriori* using the information gathered by the FFQs. Diet quality indices evaluate the overall diet quality, with a higher score usually meaning higher quality diet or higher adherence to a particular dietary pattern [5]. Thus, diet quality indices allow individuals to be categorized according to the quality of their diet. 

Useful instruments are available to assess the relationship of nutrition knowledge and attitudes with dietary intake [3]. Previous studies found a significant association between nutritional knowledge and self-diet quality. In an adult context, positive food-related attitudes have been associated with better diets measured by the Healthy Eating Index and by a higher consumption of vegetables and fruits [6]. However, evidence of the specific association of parental nutrition knowledge, and especially eating attitudes, with their children’s nutritional adequacy and diet quality is scarce. 

The aim of this study was to assess the association of certain parental nutrition knowledge and healthy-eating attitudes with their children’s nutritional adequacy and diet quality (adherence to the Mediterranean dietary pattern) in a sample of Spanish preschoolers. Findings of this investigation will help improve our comprehension of the elements that may influence children’s dietary patterns and provide valuable understanding about whether nutritional knowledge and healthy-eating attitudes are equally important.

## 2. Materials and Methods 

### 2.1. Study Aim, Design, and Setting 

The Seguimiento del Niño para un Desarrollo Óptimo (SENDO) project is a prospective, dynamic, and multipurpose pediatric cohort designed to evaluate the effect of diet and lifestyle on the health of children and adolescents. The SENDO project started in 2015. Inclusion criteria for the SENDO project included children being 4–7 years old at recruitment and who were living in Navarra (Spain). There were no exclusion criteria. The study was conducted according to the guidelines laid down in the Declaration of Helsinki and all procedures involving human subjects were approved by the ethical committee of clinical research of the Government of Navarra (Pyto2016/122). Written informed consent was obtained from all parents before the initiation of the study. This cross-sectional study was performed using data collected between 2015 and 2017.

**Figure 1:**
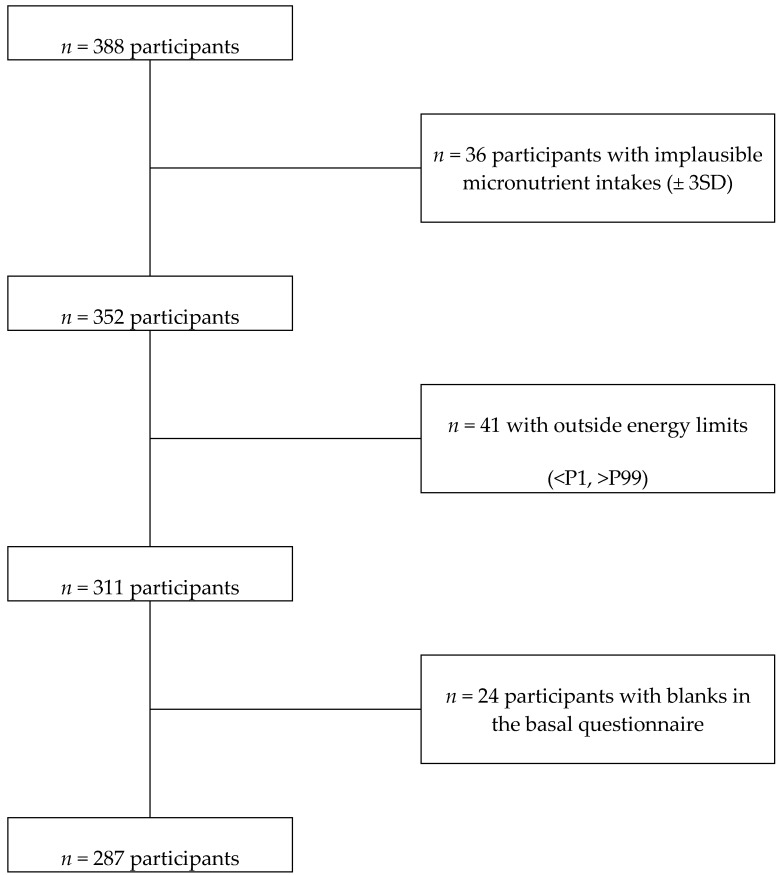
Flow-chart of participants recruited in the SENDO project, 2014–2017.

For the present study, parents who first handled the informed consent and replied to the baseline questionnaire before the end of 2017 (*n* = 388) were eligible (Figure 1). We excluded participants who reported total energy intake outside the defined limits (<P1 or >P99) or micronutrients intakes ≥3 standard deviations from the mean. We opted for a complete case analysis. Therefore, the final simple consisted of 287 participants with no missing information.

### 2.2. Exposure Assessment

The main source of retrospective information regarding children’s medical records, food intake, dietary habits, and physical activity was provided by the parents through their answers to the basal questionnaire. Anthropometric measurements (i.e., weight and height) were self-reported (reported by parents) at baseline. Participants received detailed information about how to measure children’s weight and height. This questionnaire also included a semi-quantitative FFQ, commonly used and strongly recommended, to evaluate with accuracy dietary patterns as well as diet quality.

The use of supplements was collected at baseline. Participants were asked whether they used nutritional supplements during the previous year (answer: yes vs. no). If so, they were asked to specify brand and dosage.

Information about physical activity was gathered using a specific questionnaire previously used in pediatric populations [7]. The questionnaire consisted of 14 activities, including sports, and 9 possible answers from “never” to “more than 11 hours per week”. Participants were asked how often they practiced each of the activities in the previous year. 

Parental nutrition knowledge and eating attitudes were evaluated throughout 2 different scores. Nutrition knowledge score consisted of 10 questions evaluating whether parents knew how often their children should consume 10 different food groups (Table S1) with 9 possible answers from “Never” to “≥6 times per day”. Each question was assigned 1 point if the answer was correct and 0 points if it was not based on the dietary recommendations [8]. Thus, the final score ranged from 0 to 10 points. Healthy-eating attitudes scoring consisted of 8 questions (Table S2) with 2 possible answers (Yes or No). Each affirmative answer was assigned 1 point and each negative answer was assigned 0 points. Thus, the eating attitudes score ranged from 0 to 8 points. The latter score was previously used in a cohort of Spanish university graduates [9].

Participants were categorized depending on their final score in each index in 3 groups: low (0–4 points), medium (5–6 points), or high (7–10 points) in the nutrition knowledge score, and low (0–4 points), medium (5–6 points), or high (7–8 points) in the healthy-eating attitudes score.

### 2.3. Outcome Assessment

Dietary intake was evaluated using the information gathered through a semi-quantitative FFQ consisting in 140 items with 9 possible answers, from “Never/almost never” to “≥6 times per day”. Nutrient content of each item was calculated multiplying the intake frequency by the edible portion and by the nutrient composition of the specified portion size, using data from updated Spanish food composition tables [10] and online databases. The total nutrient intake was obtained by summing the nutrient contribution of each item. 

We assessed the intake of the subsequent 19 micronutrients with known public health importance: Ca, Fe, I, Mg, Zn, Na, K, P, and Se, and vitamins B_1_, B_2_, B_3_, B_6_, B_9_, B_12_, C, A, D, and E. In order to define intake adequacy, we used the Dietary Reference Intake (DRI), particularly, the Estimated Average Requirements (EAR). The EAR of a nutrient is defined as the amount that satisfies the needs of a 50% of a homogenous healthy group.

For estimating the prevalence of inadequate micronutrient intake, we used the EAR cut-off point method. The method is based on the assessment of the proportion of individuals with nutrient intake below the EAR [11], the outcome was defined as failing to meet the recommendations in ≥3 micronutrients that represented 10–20% of the dietary micronutrients and was considered clinically relevant.

The quality of the diet was defined using KIDMED, an *a priori* defined dietary index used to evaluate the adherence to the Mediterranean dietary pattern in children and adolescents. The KIDMED index consists of 16 items (Table S3): 12 items with a score of 0 or 1, and 4 items with a score of –1 or 0. Thus, the KIDMED index ranges from 4 to 12 points [12]. According to their score, participants were considered as having poor (≤3 points), medium (4–7 points), or high adherence (≥8 points). 

### 2.4. Statistical Analysis

We compared participant’s baseline characteristics based on 3 categories of parental nutrition knowledge and the healthy-eating attitudes score. The results are presented as percentages for categorical variables and means (standard deviations) for quantitative variables. χ^2^ tests (or Fisher exact test) or ANOVA were used to assess the statistical significance of the differences of proportions and means, respectively.

Logistic regression models were used to assess the relationship of parental nutrition knowledge score and healthy-eating attitudes score with the risk of inadequate nutrient intakes. Crude odds ratios (OR) and multivariate-adjusted OR of failing to comply with ≥3 DRI were calculated. The lowest category (0–4 points) was always used as the reference group in all the analyses. Analyses were adjusted for the main known confounders regarding nutritional adequacy and diet quality based on a previous literature search on the topic. 

In additional analyses, multiple regression models were fitted to evaluate the association of parental nutrition knowledge and healthy-eating attitudes with children’s adherence to the Mediterranean dietary pattern. 

The linear trend test across the 3 categories of parental nutrition knowledge and healthy-eating attitudes were also conducted.

Analyses were carried out using Stata version 12.0 (Stata Corporation, College Station, TX, USA). All *p*-values are two tailed. Statistical significance was determined at the conventional cut-off point of *p* < 0.05.

## 3. Results

### 3.1. Recruitment and Baseline Characteristics

Participant’s socio-demographic characteristics, and food consumption and energy and nutrients intake by parental nutrition knowledge and healthy-eating attitudes are presented in Table 1 and Table 2, respectively. Parents with a higher nutrition knowledge score have children with lower body mass index (BMI), and read meat and sugar-sweetened beverage consumption, and higher intakes of vegetables, white fish, eggs, micronutrients intake, and proteins. Parents with a higher healthy-eating attitudes score had children with higher intakes of fruits, vegetables, pulses, white fish, blue fish, olive oil, and vitamin D.

### 3.2. Influence of Parental Nutritional Knowledge and Healthy-Attitudes on Food and Nutrient Intakes 

**Figure 2:**
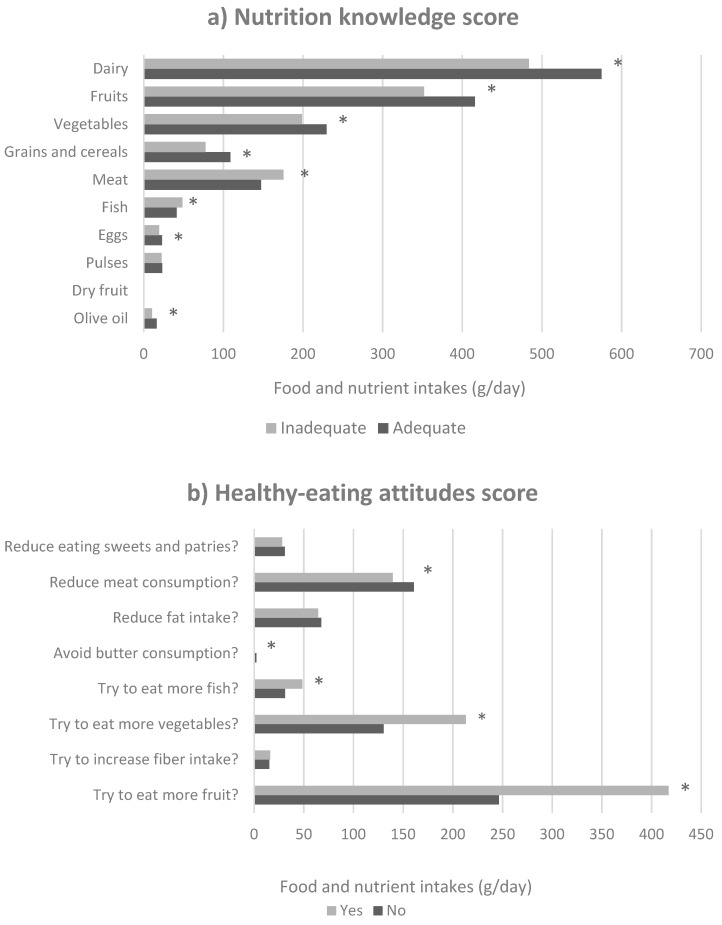
Mean food group consumption according to the positive answer in the nutrition knowledge score (**a**) and healthy-eating attitudes score (**b**); * *p* < 0.05.

Parental nutritional knowledge regarding the recommended servings per day of different food groups [8] was positively associated with adequate child mean intakes of dairy products, fruit, vegetables, grains and cereals, meat, fish, eggs and olive oil (*p* < 0.05) (Figure 2a). Similarly, healthier parental eating attitudes was associated to children lower consumption of butter and meat, as well as greater consumption of fish, vegetables, and fruit (Figure 2b).

### 3.3. Influence of Parental Nutritional Knowledge and Healthy-Eating Attitudes on Micronutrient Inadequacy

Table 3 shows the OR of failing to meet the EAR in three or more micronutrients associated to parental nutritional knowledge and healthy-eating attitudes. The highest parental category in the eating attitude score showed a significant inverse association with the risk of inadequate intake, compared to the category of reference, after adjusting for potential confounders (OR: 0.34; 95% CI: 0.12–0.95). A significant linear trend was also found (*p* < 0.05). In contrast, parental nutritional knowledge score was not associated with the risk of inadequate intake.

### 3.4. Influence of Parental Nutritional Knowledge and Healthy-Eating Attitudes on Mediterranean Adherence.

In further analyses, we found parental eating attitudes to be associated with children’s adherence to the Mediterranean dietary pattern (Table 4). Compared with the reference category, a higher healthy-eating attitudes score was marginally associated with a slightly higher score in the KIDMED index in the crude model. This association remained marginally significant after further adjustment for potential confounders (*β* coefficient: 0.34; 95% CI: 0.01–0.67). No significant association between parental nutritional knowledge and children’s adherence to the Mediterranean diet was found.

## 4. Discussion

To the best of our knowledge, this study is the first to evaluate the association of parental nutritional knowledge and healthy-eating attitudes with the micronutrient intake adequacy and diet quality among Spanish preschoolers. The results indicated that children whose parents reported healthier-eating attitudes were less likely to present micronutrient inadequacy and reported higher adherence to the Mediterranean diet. Parental nutritional knowledge was not associated with either nutritional adequacy or diet quality in their offspring, highlighting the importance of enhancing interventions to promote parental healthy eating attitudes and not simply addressing nutrition knowledge. 

Our results do not agree with previous studies that confirmed parental nutritional knowledge may affect their offspring’s diet quality [13,14,15,16,17]. Several studies have used different nutrition knowledge assessments methods [18,19], such as the nutrition knowledge questionnaire developed for adults by Parmenter and Wardle [19] used in a Japanese study or the 10-items questionnaire focusing on mis-conceptions of young children’s diet developed in a sample of Flemish preschoolers [16]. 

Although considerable discussion has focused on the influence of parental nutrition knowledge, less research has focused on the effects of parenting attitudes on their offspring’s food consumption, nutritional habits, and diet quality. Our findings agree with a recently published randomized controlled trial of a family-based behavioral nutrition intervention [20] in children with type 1 diabetes that concluded that parents’ diet-related attitudes and beliefs were linked to their children’s diet quality, remarking on the essential role of parental psychosocial factors. 

Thus, our results are not surprising, as it is coherent and plausible to assume healthy-eating attitudes to be important determinants of children’s eating behavior, even to a greater extent than parental nutritional knowledge. Attitudes seem to be based on family influence, experiences, knowledge, and norms imposed by the environment [21]. Parents with greater nutritional knowledge may choose healthier options by interpreting nutritional information and labeling, but knowledge seems useless if it is not put into practice or it is not accompanied by healthy attitudes. 

In our study, we also observed that children whose parents answered positively to the questions in the healthy-eating attitudes index consumed more fruit, vegetables, and fish and less butter and meat. Furthermore, children whose parents demonstrated a knowledge of the dietary recommended intakes of olive oil, eggs, fish, meat, grains and cereals, vegetables, fruit, and dairy products consumed appropriate amounts of them. These findings are consistent with previous studies that identified family environment, education, eating attitudes, nutritional knowledge, availability, and family norms are positively associated with children’s and adolescents’ consumption of fruit and vegetables [22,23,24,25] as well as fish [26], and negatively associated with fat consumption [27].

Despite these significant results, our study has limitations. First, FFQs may not be the best dietary assessment method for identifying nutrient inadequacy because they include a limited number of foods [28]. FFQs, being self-administered, might lead to measurement error. Nevertheless, FFQs have been used in previous epidemiological studies to assess micronutrient inadequate intake [29] based on the idea that FFQs are the most efficient and feasible instruments to assess usual dietary intake. The FFQ used in this study was large (140 items), comprising most of the food items commonly consumed by Spanish children, and included the possibility of adding three extra food items. The potential random error due to a suboptimal classification of the participant according to their micronutrient intake would be non-differential; thus, the association would more likely be biased toward the null, making it more difficult to find statistically significant results. Second, to calculate micronutrient intakes from the information collected through the FFQs, we used Spanish food composition tables [10] and online databases, which might be slightly different to those found in other sources. Third, we did not consider micronutrients from supplements, fortified foods, or medication, which might have resulted in an underestimation of the real intakes. Fourth, our questionnaire, including the FFQ, needs to be validated in further studies. Nevertheless, the healthy-eating attitudes score was previously used in a cohort of Spanish university graduates [9]. Fifth, despite the significant results, we acknowledge the change in the KIDMED index was small. Thus, further research is needed to assess the real magnitude of this association. Sixth, our sample was homogenous, with a large part of participants being Caucasian and highly-educated parents. However, previous studies have shown that, for causal inference, representativeness of the sample is not necessary, and may be detrimental when measurement tools involve some difficulty or require an important collaboration from participants, as it is the case with FFQs [30,31]. Lastly, we used a cross-sectional design. Thus, further research is needed, preferably in the form of prospective studies, before causality can be inferred.

Our study has several strengths. We used EAR as the cut-off point to assess adequate nutrient intakes, which is widely recommended by the Institute of Medicine [11] and has been previously used by the European Food Safety Authority [32]. Our study adds to the current knowledge because we defined the outcome as failing to meet the recommended dietary intakes of three or more micronutrients. To the best of our knowledge, no previous study has defined this outcome. However, growth retardation and several adverse outcomes throughout life might be caused by micronutrient deficiencies. We add to this topic since micronutrient intake adequacy has not been studied in detail in preschool children [33]. 

## 5. Conclusions

In conclusion, our findings are valuable as they highlight the importance of recognizing and better understanding the role of parents, particularly their own eating attitudes, on their children’s diet quality and on their long-term health. Our results suggest that food knowledge alone may not be enough and that the implementation of parental behavioral-focused programs may be more efficient than usual nutrition education programs in order to improve children’s diet quality. 

## Figures and Tables

**Table 1:** Baseline main characteristics of the 287 participants of the SENDO project according to nutrition knowledge and healthy-eating attitudes score (Mean values and standard deviations or number of participants and percentages).

	Nutrition Knowledge Score				Healthy-Eating Attitudes Score			
	Low	Medium	High		Low	Medium	High	
	0–4	5–6	7–10		0–4	5–6	7–8	
**Children Characteristics**				***p* value**				***p* value**
*n* (%)	87 (30.31)	122 (42.51)	78 (27.18)		69 (24.04)	111 (38.68)	107 (37.28)	
Age (years)	7.2 (1.81)	6.89 (1.69)	6.56 (1.63)	0.063	6.62 (1.74)	6.93 (1.67)	7.03 (1.76)	0.299
Sex (%)								
Girls	56.3	44.3	48.7		56.5	47.7	45.8	
BMI (kg/m^2^)	15.9 (1.7)	15.8 (1.5)	15.3 (1.5)	0.035	15.7 (1.4)	15.5 (1.6)	15.8 (1.6)	0.522
Waist /Height ratio	0.47 (0.05)	0.48 (0.05)	0.48 (0.06)	0.644	0.49 (0.05)	0.47 (0.05)	0.48 (0.06)	0.055
Supplement use (%)	4.65	4.13	3.9	0.970	7.25	1.82	4.76	0.201
KIDMED test	5.86 (1.31)	5.87 (0.97)	6.1 (0.95)	0.258	5.74 (1.08)	5.89 (1.19)	6.09 (0.95)	0.093
Physical activity (MET-h/week)	34.1 (27.8)	42.9 (31.3)	37.4 (22.6)	0.075	36.2 (26.1)	38 (27.2)	41.2 (30.7)	
Leisure activity (%) ^1^	50.6	54.1	56.4	0.748	49.3	60.4	49.5	0.195
Eating at home (%) ^2^	71.3	77.9	85.9	0.076	73.9	77.5	81.3	0.503
Eating with someone (%) ^3^	79.3	86.1	80.8	0.396	79.7	82.9	84.1	0.750
**Parental Characteristics**								
Age (years)								
Mother	39.7 (4.5)	39.2 (3.2)	39 (3.2)	0.449	38.9 (4.2)	39.5 (3.6)	39.3 (3.3)	0.531
Father	37.6 (4)	37.3 (3.2)	37.1 (3.1)	0.548	36.7 (3.5)	37.9 (3.6)	37.2 (3.2)	0.072
Large family (%) ^4^	35.6	31.1	28.2	0.583				0.289
Education (%)								
University	50.6	54.9	66.7	0.098	56.5	58.6	55.1	0.877
Do you consider your								
children’s weight as high? (%)	1.15	0.82	0	0.433	0	1.8	0	0.699
Fixed eating schedule (%)	94.3	93.4	93.6	0.970	94.2	92.8	94.4	0.873
Snacking (%)	6.9	7.38	7.69	0.980	8.7	7.21	6.54	0.865

^1^ Leisure activity includes: extra-curriculum activities other than sports (music, English lessons…); ^2^ Eating at home (%): yes/no; ^3^ Eating with someone (%): yes/no; ^4^ Large family composed of ≥3 children. MET; Metabolic Equivalent.

**Table 2:** Mean food and nutrients intakes among the 287 participants of the SENDO project according to the nutrition knowledge score and healthy-eating attitudes score (Mean values and standard deviations).

	Nutrition Knowledge Score				Healthy-Eating Attitudes Score			
	Low	Medium	High		Low	Medium	High	
	0–4	5–6	7–10		0–4	5–6	7–8	
**Food Group**				***p* value**				***p* value**
Fruit (g/day)	368 (190)	413 (199)	409 (176)	0.212	364 (174)	384 (186)	435 (201)	0.032
Vegetables (g/day)	180 (91)	218 (111)	220 (97)	0.013	190 (116)	198 (80)	229 (110)	0.022
Pulses (g/day)	23.5 (15.3)	23 (11.1)	22.6 (6.7)	0.896	22.5 (13.1)	21 (8.3)	25.5 (13)	0.014
Daily (g/day)	503 (218)	563 (231)	559 (195)	0.114	540 (223)	532 (211)	557 (225)	0.698
White meat (g/day)	39.8 (20.9)	43.6 (19.4)	40.4 (16.4)	0.306	40.2 (18.1)	39.4 (17)	44.8 (21.5)	0.090
Red meat (g/day)	111 (43)	122 (50)	105 (39)	0.035	123 (48)	113 (36)	109 (53)	0.136
White fish (g/day)	26.8 (15.1)	31.6 (16.5)	33 (14.2)	0.023	26.4 (15.7)	29.7 (16.3)	34 (14.3)	0.005
Blue fish (g/day)	11.5 (9.3)	13 (13.5)	15.2 (13.2)	0.152	9.6 (9.6)	12.5 (12.6)	16.1 (13)	0.002
Olive oil (g/day)	12.7 (13.1)	11.5 (11.4)	13.9 (13.7)	0.424	12.6 (11.4)	10.4 (11.2)	14.6 (14.21)	0.045
Bakery (g/day)	27.7 (35)	30.2 (23.2)	27 (19.8)	0.652	30.2 (38)	27.6 (21.4)	28.6 (22.2)	0.822
Eggs (g/day)	18 (10)	22 (14.5)	25.7 (15.8)	0.002	20.8 (8.1)	21.5 (15.6)	22.7 (15.2)	0.650
Beverage (g/day)	7.6 (14.81)	4.6 (8.1)	3.5 (6.4)	0.024	7.8 (13.3)	4.2 (7.4)	4.6 (10.8)	0.054
Sweets (g/day)	17.1 (14.2)	14.8 (9.5)	13.8 (13.9)	0.199	15.8 (12.1)	15.2 (11)	14.9 (13.8)	0.894
**Nutrients**								
Energy (kcal/day)	1700 (409)	1787 (432)	1775 (365)	0.286	1753 (395)	1708 (361)	1811 (458)	0.175
HC (% total energy)	47.8 (6.3)	46.4 (5.3)	47.9 (4.9)	0.109	47 (5.7)	46.6 (5.6)	48.1 (5.4)	0.125
Proteins (% total energy)	19.2 (2.3)	19.8 (2.6)	19.1 (1.7)	0.026	19.3 (2.7)	19.7 (2.5)	19.2 (1.9)	0.323
Fat (% total energy)	33.1 (5.6)	33.7 (4.4)	33 (4.6)	0.533	33.7 (4.9)	33.7 (4.6)	32.7 (5)	0.218
Ca (mg/day)	1102 (344)	1242 (441)	1295 (449)	0.008	1184 (380)	1198 (421)	1251 (449)	0.520
Fe (mg/day)	9.1 (6)	11.1 (7.5)	12.6 (7.9)	0.006	11 (6.9)	10.4 (7.2)	11.3 (7.8)	0.680
I (µg/day)	92 (47)	111 (53)	121 (61)	0.002	108 (53)	104 (53)	112 (57)	0.618
Mg (mg/day)	224 (70)	263 (95)	282 (111)	0.000	256 (85)	246 (88)	268 (109)	0.241
Zn (mg/day)	5.7 (2.4)	6.9 (3.4)	7.4 (3.9)	0.002	6.7 (3.1)	6.5 (3.1)	6.9 (3.6)	0.764
Na (mg/day)	2477 (1095)	3007 (1361)	3324 (1310)	0.000	3175 (1298)	2841 (1321)	2871 (1296)	0.207
K (mg/day)	2401 (1170)	2907 (1380)	3177 (1566)	0.001	2798 (1269)	2686 (1354)	2992 (1526)	0.269
P (mg/day)	1024 (335)	1224 (447)	1285 (502)	0.000	1171 (389)	1150 (436)	1217 (485)	0.534
Se (µg/day)	42.9 (35.4)	54.4 (43.8)	63.1 (47.6)	0.01	55.1 (41.8)	51.2 (42.1)	54.3 (45.2)	0.802
Vitamin B_1_ (mg/day)	0.8 (0.5)	1.02 (0.62)	1.1 (0.7)	0.004	1.00 (0.57)	0.9 (0.6)	0.9 (0.6)	0.929
Vitamin B_2_ (mg/day)	1.3 (0.4)	1.6 (0.6)	1.66 (0.62)	0.000	1.5 (0.5)	1.5 (0.6)	1.6 (0.6)	0.825
Vitamin B_3_ (mg/day)	16.0 (9.7)	23.6 (12.8)	27.6 (24.7)	0.000	22.6 (12.9)	21.9 (11.1)	22.7 (11.4))	0.948
Vitamin B_6_ (mg/day)	1.2 (0.7)	1.5 (0.9)	1.6 (0.9)	0.001	1.4 (0.8)	1.4 (0.94)	1.5 (0.9)	0.810
Vitamin B_9_ (µg/day)	178 (90)	220 (123)	237 (123)	0.002	205 (106)	208 (123)	221 (115)	0.573
Vitamin B_12_ (µg/day)	2.3 (1.1)	3.07 (1.61)	3.4 (1.7)	0.000	2.93 (1.55)	2.8 (1.6)	2.9 (1.6)	0.902
Vitamin C (mg/day)	79.9 (47.9)	100.4 (50.1)	107.3 (52.3)	0.001	89.3 (44.8)	94.2 (52.8)	102.4 (52.8)	0.224
Vitamin A (µg/day)	465 (272)	603 (462)	680 (404)	0.002	583 (427)	569 (435)	595 (357)	0.894
Vitamin D (µg/day)	1.3 (1.3)	1.5 (1.4)	2.15 (1.92)	0.001	1.4 (1.34)	1.4 (1.4)	1.95 (1.83)	0.024
Vitamin E (µg/day)	14.7 (21)	16.8 (22.9)	21.9 (24.8)	0.121	17.6 (22.9)	15.7 (22)	19.4 (24.1)	0.504

**Table 3:** Risk of nutrient inadequacy (failing to meet the estimated average requirements (EAR) in three or more micronutrients) associated to parental nutrition knowledge and healthy-eating attitudes score in the participants of the SENDO project.

	Nutrition Knowledge Score						
	Low	Medium			High		*p*
		OR	95% CI		OR	95% CI	
*N*	65	83			47		
% intake failing to meet ≥3 EAR	33.33	42.56			24.10		
Crude	1 (Ref.)	0.72	0.39, 1.33		0.51	0.26, 1.00	0.051
Multivariable 1	1 (Ref.)	0.84	0.36, 1.98		0.70	0.28, 1.75	0.449
Multivariable 2	1 (Ref.)	0.80	0.34, 1.91		0.63	0.25, 1.60	0.337
Multivariable 3	1 (Ref.)	1.34	0.50, 3.59		1.13	0.40, 3.23	0.961
Multivariable 4 ^1^	1 (Ref.)	1.45	0.54, 3.90		1.19	0.41, 3.48	0.908
	**Healthy-Eating Attitudes Score**						
	**Low**	**Medium**			**High**		***p***
		**OR**	**95% CI**		**OR**	**95% CI**	
*N*	49	77			69		
% intake failing to meet ≥3 EAR	25.13	39.49			35.38		
Crude	1 (Ref.)	0.92	0.48, 1.79		0.74	0.39, 1.42	0.322
Multivariable 1	1 (Ref.)	0.60	0.25, 1.46		0.35	0.14, 0.88	0.026
Multivariable 2	1 (Ref.)	0.56	0.23, 1.38		0.34	0.14, 0.86	0.027
Multivariable 3	1 (Ref.)	0.63	0.23, 1.69		0.34	0.12, 0.93	0.034
Multivariable 4 ^2^	1 (Ref.)	0.63	0.23, 1.72		0.34	0.12, 0.95	0.037

Ref.: reference. Multivariable 1: adjusted for age and sex. Multivariable 2: additionally adjusted for BMI. Multivariable 3: additionally adjusted for energy intake, KIDMED index and parental education level. Multivariable 4 ^1^: additionally adjusted for physical activity (MET- hour/week) and healthy-eating attitudes. Multivariable 4 ^2^: additionally adjusted for physical activity (MET- hour/week) and nutrition knowledge.

**Table 4:** Multivariate regression coefficients (*β* coefficient and 95% confidence intervals) for the association of parental nutrition knowledge and healthy-eating attitudes score with children’s score in the KIDMED test.

Nutrition Knowledge Score							
KIDMED	Low	Medium			High		
		*β*	95% CI	*p*	*β*	95% CI	*p*
Crude	0 (Ref.)	0.01	−0.29, 0.31	0.964	0.24	−0.09, 0.57	0.154
Multivariable 1	0 (Ref.)	0.02	−0.28, 0.32	0.903	0.27	−0.07, 0.60	0.119
Multivariable 2	0 (Ref.)	0.02	−0.28, 0.32	0.897	0.28	−0.06, 0.62	0.110
Multivariable 3	0 (Ref.)	−0.01	−0.31, 0.30	0.971	0.23	−0.12, 0.57	0.195
Multivariable 4 ^1^	0 (Ref.)	−0.01	−0.31, 0.30	0.969	0.18	−0.16, 0.53	0.293
**Healthy-Eating Attitudes Score**							
**KIDMED**	**Low**	**Medium**			**High**		
		***β***	**95% CI**	***p***	***β***	**95% CI**	***p***
Crude	0 (Ref.)	0.15	−0.17, 0.48	0.355	0.35	0.03, 0.68	0.034
Multivariable 1	0 (Ref.)	0.15	−0.18, 0.47	0.381	0.35	0.01, 0.68	0.041
Multivariable 2	0 (Ref.)	0.15	−0.18, 0.48	0.379	0.35	0.01, 0.68	0.041
Multivariable 3	0 (Ref.)	0.15	−0.18, 0.48	0.369	0.36	0.03, 0.69	0.033
Multivariable 4 ^2^	0 (Ref.)	0.15	−0.18, 0.48	0.361	0.34	0.01, 0.67	0.045

Ref.: reference. Multivariable 1: adjusted for age and sex. Multivariable 2: additionally adjusted for BMI. Multivariable 3: additionally adjusted for energy intake, KIDMED index and parental education level. Multivariable 4 ^1^: additionally adjusted for physical activity (MET: hour/week) and healthy-eating attitudes. Multivariable 4 ^2^: additionally adjusted for physical activity (MET- hour/week) and nutrition knowledge.

## Supplementary Materials

The following are available online at http://www.mdpi.com/2072-6643/10/12/1875/s1, Table S1: Criteria used to calculate nutrition knowledge score. Table S2: Criteria used to calculate healthy-eating attitudes score. Table S3: KIDMED test with the original punctuation.
