# Supplementary material for: Influence of Parental Healthy-Eating Attitudes and Nutritional Knowledge on Nutritional Adequacy and Diet Quality among Preschoolers: The SENDO Project
Source: Nutrients. 2018 Dec 3;10(12):1875. doi: 10.3390/nu10121875 (PMC6316633; doi:10.3390/nu10121875)
Supplement: Supplementary file 1 [file nutrients-10-01875-s001.pdf]

Table S1. Criteria used to calculate nutrition knowledge score.

How often do you think your child should consume these foods to follow a healthy diet?	Never or almost never	1–3 times/month	1/week	2–4 times/week	5–6 times/week	1/day	2–3 times/day	4–6 times/day	More 6 times/day
Dairy	0	0	0	0	0	0	1	0	0
Fruit	0	0	0	0	0	0	1	0	0
Vegetables	0	0	0	0	0	0	1	0	0
Cereals and grains	0	0	0	0	0	0	0	1	0
Meat	0	0	0	1	0	0	0	0	0
Fish	0	0	0	1	0	0	0	0	0
Eggs	0	0	0	1	0	0	0	0	0
Pulses	0	0	0	1	0	0	0	0	0
Nuts	0	0	0	1	1	0	0	0	0
Olive oil	0	0	0	0	0	0	1	1	0

Table S2. Criteria used to calculate healthy-eating attitudes score.

I try my child to eat more …	NO	YES
Fruit?	0	1
Fiber?	0	1
Vegetables?	0	1
Fish?	0	1
Avoid butter consumption?	0	1
Reduce fat intake?	0	1
Reduce meat consumption?	0	1
Reduce eating sweets and pastries?	0	1
Total (min/max)	0	8

Table S3. KIDMED test with the original punctuation.

KIDMED test (Mediterranean Diet Quality Index)	Scoring
Fruit or fruit juice daily	+1
Second serving of fruit daily	+1
Fresh or cooked vegetables daily	+1
Fresh or cooked vegetables > 1/day	+1
Regular fish consumption (at least 2–3/week)	+1
>1/week fast-food (hamburger) restaurant	-1
Pulses > 1/week	+1
Pasta or rice almost daily (≥5 days/week)	+1
Cereal or cereal product for breakfast	+1
Regular nut consumption (at least 2–3/week)	+1
Use of olive oil at home	+1
No breakfast	-1
Dairy product for breakfast	+1
Commercially baked goods or pastries for breakfast	-1
Two yoghurts and/or 40 g cheese daily	+1
Sweets and candy several times a day	-1
